# Comparative evaluation of the immunodominant proteins of *Brucella abortus* for the diagnosis of cattle brucellosis

**DOI:** 10.14202/vetworld.2021.803-812

**Published:** 2021-03-30

**Authors:** Mohandoss Nagalingam, Thaslim J. Basheer, Vinayagamurthy Balamurugan, Rajeswari Shome, S. Sowjanya Kumari, G. B. Manjunatha Reddy, Bibek Ranjan Shome, Habibur Rahman, Parimal Roy, J. Joseph Kingston, R. K. Gandham

**Affiliations:** 1 ICAR-National Institute of Veterinary Epidemiology and Disease Informatics, Bengaluru, Karnataka, India; 2 International Livestock Research Institute, New Delhi, India; 3 Defense Food Research Laboratory, Mysore, Karnataka, India; 4 National Institute of Animal Biotechnology, Hyderabad, Telangana, India

**Keywords:** *Brucella abortus*, *Brucella* lumazine synthase, *Brucella* protein26, cattle brucellosis, Cu-Zn superoxide dismutase, *Yersinia enterocolitica* O:9

## Abstract

**Background and Aim:**

The present serodiagnosis of brucellosis in livestock is based on the whole cell or smooth lipopolysaccharide of the *Brucella* organism in which specificity is hampered by the cross-reactivity, especially with the antibodies against *Yersinia enterocolitica* O:9 organism. The problem can be addressed by screening for better immunodominant antigens. Hence, the present study was undertaken to screen protein antigens of *Brucella abortus* for their diagnostic potential in cattle brucellosis.

**Materials and Methods:**

Protein antigens of *B. abortus* (n=10) non-reactive to antibodies against *Y. enterocolitica* O:9 were selected, expressed in *Escherichia coli*, assessed the reactivity of expressed recombinant proteins by Western blot, standardized indirect-enzyme-linked immunosorbent assay (ELISA) for detecting *Brucella* antibodies in cattle serum, and comparative evaluation was done.

**Results:**

All the selected protein antigens were expressed and in the Western blot with *Brucella* antibodies positive cattle serum, six recombinant (*Brucella* protein 26 [BP26], Cu-Zn Superoxide dismutase [SodC], *B. abortus* I-1885, Serine protease, Bacterioferritin, and *Brucella* Lumazine Synthase [BLS]) proteins showed reaction whereas none of the proteins showed reactivity with *Brucella* negative cattle serum. ELISA has been done using known *Brucella* positive and negative cattle sera samples (n=113 each) in which the performance of recombinant proteins in diagnosing brucellosis was in the order of BP26 > BLS > SodC followed by rest of the proteins. BP26 based ELISA was found to be better with area under the curve as 0.953, and diagnostic sensitivity, diagnostic specificity, and Youden’s index of 90.27%, 95.58%, and 0.8584, respectively, with the excellent agreement (k=0.85).

**Conclusion:**

BP26 could be a potential diagnostic antigen among the immunodominant proteins of *B. abortus* in ruling out *Y. enterocolitica* O:9 infection while diagnosing brucellosis in cattle herds.

## Introduction

Brucellosis is a multifaceted zoonotic disease with significant animal and human health impact, caused by facultative, intracellular bacteria of genus *Brucella*, which comprises six main species, namely *Brucella abortus, Brucella melitensis, Brucella suis, Brucella ovis, Brucella canis, and Brucella neotomae*, and other recently identified species include *Brucella pinnipedialis, Brucella ceti, Brucella microti, Brucella inopinata, Brucella papionis*, and *Brucella vulpis* [1]. The disease is usually represented by abortion, reduced fertility, and reduced milk production in ruminants. The common species involved in causing brucellosis in cattle is *B. abortus*.

Since the discovery of the brucellosis, the conventional serological tests, namely, Rose Bengal plate test (RBPT), standard tube agglutination test (SAT), enzyme-linked immunosorbent assay (ELISA), and complement fixation tests which utilize the whole *Brucella* cell and/or its smooth lipopolysaccharide (S-LPS) fractions are being used in diagnosis [2]. Cross-reactivity arises due to the resemblance of *Brucella* O-polysaccharide, a component of LPS with corresponding epitopes of *Yersinia enterocolitica* O:9, *Salmonella urbana* group N, *Vibrio cholerae, Francisella tularensis*, *Escherichia coli* O157, and *Stenotrophomonas maltophilia* [3]. In particular, serological interference induced by *Y. enterocolitica* O:9 has complicated the eradication of brucellosis in some European countries such as France, Belgium, United Kingdom [4,5] with the prevalence of *Y. enterocolitica* ranging from 18% to 58% in cattle [6]. In European Union, 15% of the herds in regions free from brucellosis were infected with *Y. enterocolitica* O:9 [7]. After 1990, the isolation of *Y. enterocolitica* O:9 from cattle has become a regular phenomenon not only from European Union but also from other parts of the world including New Zealand [8]. As diagnosis plays a key role in disease control/eradication programs, there is a strong need for tests that can avoid cross-reactivity, in particular against *Y. enterocolitica* O:9. This can be overcome by screening antigens other than surface antigens like S-LPS. Various proteins of *Brucella* spp. have been investigated for their use as a diagnostic antigen, replacing S-LPS either in the form of the purified native protein(s) or synthesized recombinant protein(s). Different proteins were reported from different research groups for their capacity in diagnosis; however, still, there is a scope for further screening.

The present study was aimed to screen better protein antigens for diagnosing cattle brucellosis which is void of cross-reactivity, especially against antibodies of *Y. enterocolitica* O:9. ELISA was done with these antigens and different cattle sera samples and compared for better performance. To the best of our knowledge, it is the first study comparing ten recombinant proteins that are non-reactive to *Y. enterocolitica* O:9.

## Materials and Methods

### Ethical approval

The Institute Biosafety committee approval (F. No. 6-52/NIVEDI/Biosafety/2016/07-19 dtd.11.12.2017) was obtained for the work.

### Study period and location

The work was conducted at ICAR-NIVEDI from January 2016 to March 2018. The cattle sera were collected from Karnataka, India.

### Bacterial strains, vectors, and serum

*B. abortus* Strain 99 (NCTC 11363) procured from Indian Council of Agricultural Research Institute (ICAR)-Indian Veterinary Research Institute, Izatnagar, India, was used to obtaining deoxyribonucleic acid (DNA). HI-Control 10G and HI-Control BL21 (DE3) chemically competent cells with pETite N-His Kan Vector (M/s Lucigen, USA) were used in this study for expression of proteins in *E. coli* system. Hyperimmune serum raised against *B. abortus* S99 and *Y. enterocolitica* O:9 in rabbit, rabbit serum tested negative for *Brucella* antibodies, and serum samples collected from different cattle were used in this study. The reference DNA sequence of *B. abortus* biovar 1 strain 9-941 was used for designing primers, comparison of amplified sequences, etc., The NCBI reference number for chromosome 1 is NC_006932 (2124241 bp) and that of chromosome 2 (1162204 bp) is NC_006933.

### Selection of immunodominant proteins/protein antigens for expression

The immunodominant proteins were identified based on a literature survey concerning their reactivity with *Brucella* positive serum and non-reactivity with *Y. enterocolitica* O:9 positive serum. They include Cu-Zn superoxide dismutase (SodC), Serine protease, BAB1-1885, Twin arginine translocation pathway signal sequence domain-containing protein (Twin arginine), *Brucella* protein26 (BP26), Solute-binding family 5 protein, Leu/Ile/Val-binding family protein, Branched-chain amino-acid ABC transporter substrate-binding protein, Thiamine transporter binding protein, Invasion protein B (InvB), *Brucella* lumazine synthase (BLS), bacterioferritin (Bfr), malate dehydrogenase (Mdh), VirB12, and Aldehyde dehydrogenase. The selected proteins are outer membrane or periplasmic except for BLS, Bfr, Mdh, VirB12, and Aldehyde dehydrogenase which are cytoplasmic proteins. These identified proteins were further checked through BLASTP for amino acid sequence identity and those showing ≤60% with other cross-reacting pathogens were selected for further expression (Table-1).

**Table-1:** Immunodominant proteins of *Brucella* *abortus* selected for expression in *Escherichia coli.*

Protein/Gene	Chromosome no./locus tag	Accession No.	Start position	End position	Base pairs (bp)	Amino acid	Signal protein	Amino acids without signal protein if any	Estimated MW without signal protein if any (KDa)
Twin arginine (BAB1_0521)/*twin arginine*	1/BRUAB_RS02470	NC_006932	516063	516716	654	217	1-29	188	20.68
Invasion protein B (InvB)/*invB*	2/BRUAB_RS13540	NC_006933	663014	663631	618	205	1-29	176	19.36
Thiamine transporter substrate binding subunit (ThiB)/*thiB*	1/BRUAB_RS08405	NC_006932	1706105	1707109	1005	334	1-23	311	34.21
BP26 (OMP28)/*bp26*	1/BRUAB_RS07100	NC_006932	1447720	1448472	753	250	1-28	222	24.72
Cu/Zn superoxide dismutase (SodC)/*sodC*	2/BRUAB_RS12940	NC_006933	534081	534602	522	173	1-19	154	16.94
BAB1-1885/*bab1-1885*	1/BRUAB_RS08980	NC_006932	1836219	1837595	1377	458	-	457	50.27
Serine protease (htrA)/*serine protease*	1/BRUAB_RS03035	NC_006932	621517	623058	1542	513	1-25	488	53.68
Bacterioferritin/*bfr*	2/BRUAB_RS13595	NC_006933	673947	674432	486	161	-	160	17.60
VirB12 (BruAb2_0058)/*virB12*	2/BRUAB_RS10670	NC_006933	559345	56452	519	172	-	171	18.81
Brucella Lumazine Synthase (BLS)/*bls*	1/BRUAB_RS03755	NC_006932	776191	776664	474	157	-	156	17.16

### Expression of immunodominant proteins

The primers were designed for the selected gene sequences to suit enzyme-free cloning in the pETite N-His Kan vector (Table-2). The respective gene sequences of selected proteins were amplified by PCR with reaction mixture comprising of 10× PCR buffer-5 μL; forward and reverse primers 10 mM each 1 μL; dNTP (10 mM)-1 μL; *B. abortus* S99 template DNA 2 μL (94 ng); high fidelity Taq polymerase 0.5 μL (M/s. Thermo Scientific, USA); and nuclease-free water were added to make up 50 μL. PCR was performed in the thermal cycler (M/s. Eppendorf, Germany) with the cycling conditions as mentioned in Table-2. The amplified product was run on 1.5% agarose gel containing ethidium bromide (0.5 mg/μL), visualized and documented in UV trans-illuminator.

**Table-2:** List of designed oligonucleotides for amplification of gene sequences coding for selected immunodominant proteins of *B. abortus* and their size along with PCR cycling conditions.

Gene	Primer*	Sequence (5’- 3’)^#^	Primer start position in the gene (bp)	Primer end position in the gene	Amplicon size	Cycling conditions for PCR amplification
*twin arginine*	F	**CAT CAT CAC CAC CAT CAC** CAG CAA CAC GCC CCG GAA G	88	651	564	**1. Initial denaturation** 94ºC for 3 min **2. Cycles (n = 35)** i. *Denaturation:* 94ºC for 1 min ii. *Annealing:* 50-60ºC for 1 min. with increment of 1ºC per cycle iii. *Extension:* 72ºC for 1 min. for genes less than 1 Kb and 72ºC for 2 min. for genes more than 1 Kb. 3. **Final extension** 72ºC for 10 min
	R	**GTG GCG GCC GCT CTA TTA** AAG AGC GCT GTC GAT GAA TCC				
*invB*	F	**CAT CAT CAC CAC CAT CAC** CAG CAG CCG CCG CAG GGT T	88	615	528	
	R	**GTG GCG GCC GCT CTA TTA** TTT GGC AGC GCC TTT TGC CTT				
*thiB*	F	**CAT CAT CAC CAC CAT CAC** AAG GAC AAG CTT ACT ATC TAT AC	70	1002	933	
	R	**GTG GCG GCC GCT CTA TTA** TCT GCT GGT GGC TGC CAG C				
*bp26*	F	**CAT CAT CAC CAC CAT CAC** CAG GAG AAT CAG ATG ACG ACG	85	750	666	
	R	**GTG GCG GCC GCT CTA TTA** CTT GAT TTC AAA AAC GAC ATT GAC				
*sodC*	F	**CAT CAT CAC CAC CAT CAC** GAA AGC ACG ACG GTA AAA ATG TAT G	58	519	462	
	R	**GTG GCG GCC GCT CTA TTA** TTC GAT CAC GCC GCA GGC AAA AC				
*BAB-1885*	F	**CAT CAT CAC CAC CAT CAC** GCG AAA TCC GGC ACC CCG	4	1374	1371	
	R	**GTG GCG GCC GCT CTA TTA** CTG ACC GGA AGA GGC CGG				
*serine protease*	F	**CAT CAT CAC CAC CAT CAC** TTC GTC GTA ACC GGC CCG	76	1539	1464	
	R	**GTG GCG GCC GCT CTA TTA** TTC CTG ATT GAT CGG CAG CG				
	R	**GTG GCG GCC GCT CTA TTA** TTT CAG CGA CGG AGC AAT AC				
*virB12*	F	**CAT CAT CAC CAC CAT CAC** CGC ACA TTG GTT ATG GTC GC	4	516	513	
	R	**GTG GCG GCC GCT CTA TTA** CTT GCG TAA AAT TTC GAT ATC C				
*bfr*	F	**CAT CAT CAC CAC CAT CAC** AAA GGC GAA CCA AAG GTC ATC	4	483	480	
	R	**GTG GCG GCC GCT CTA TTA** CTC AGC TTC GTC GGC GGG				
*bls*	F	**CAT CAT CAC CAC CAT CAC** AAC CAA AGC TGT CCG AAC AAG AC	4	474	471	
	R	**GTG GCG GCC GCT CTA TTA** GAC AAG CGC GGC GAT GCG				

* F = Forward, R = Reverse,

# Nucleotides in bold bind to the vector for enzyme free cloning

The PCR amplicons were individually transformed with pETite N-His Kan vector into HI-Control 10G chemically competent cells, respectively, and recombinant colonies obtained were screened by colony PCR. The plasmids were extracted from the positive clones and double pass sequencing of plasmid DNA was carried out commercially (M/s. Eurofins, India) by vector-specific primers. After confirmation by sequencing, pETite N-His Kan vectors containing gene insert were individually transformed into HI-Control BL21 (DE3) and confirmed by colony PCR. The recombinant pETite N-His Kan BL21 clones were induced with IPTG for expression. The expression was optimized for the incubation time (1-5 h after induction) and IPTG concentration (0.5-2.0 mM). The expressed proteins were characterized by SDS-PAGE and purified with Ni-NTA purification system and subsequently dialyzed. The Western blot was carried out with rabbit negative and hyperimmune sera raised against *B. abortus* S99 antigen, *Y. enterocolitica* O:9 antigen, and cattle sera (positive and negative for *Brucella* antibodies confirmed by RBPT and Svanovir Indirect ELISA [Svanova, Sweden] for brucellosis).

### Selection of *Brucella* antibodies positive and negative cattle sera

A total of 573 cattle sera were collected both from brucellosis infected (abortions were common and *B. abortus* etiology established earlier) and non-infected farms (No abortion and *B. abortus* etiology absent) and none of the animals were vaccinated for brucellosis. The samples were screened by RBPT [9], Svanovir Indirect ELISA kit for brucellosis (Svanova, Sweden), and Svanovir Competitive ELISA kit for brucellosis (Svanova) following manufacturer instructions, and out of that 114 samples were positive by all the three tests/assays and 322 samples were negative by all the tests for *Brucella* antibodies. For establishing diagnostic sensitivity (DSe) and diagnostic specificity (DSp), a sample size of 113 positive and 113 negative sera from the above samples by all tests was selected for estimated DSe and DSp as 92%, with an error margin of 5% at 95% confidence interval [10]. Further, the *Brucella* antibody titers were determined by the STAT/SAT [9]. The titer of selected positive sera from cattle ranged from 1 in 10 to 1 in 640 and hyperimmune serum from rabbit was having more than 1 in 2560 whereas all the negative sera were not having any titer. The negative samples were selected only from non-infected farms where no single positive reactor was present.

### Standardization of recombinant protein Indirect ELISA (I-ELISA)

I-ELISA was standardized for antigen concentration and serum dilution by checkerboard titration using *Brucella* positive and negative serum samples. The recombinant proteins which have reacted in Western blot with cattle serum positive for *Brucella* antibodies were subjected individually for checkerboard titration for antigen concentration and serum dilution [11]. ELISA was done according to standard ELISA protocol. The recombinant proteins as antigen(s) were doubly diluted in a 96 well ELISA plate ranging from 16 mg to 7.8 ng along the row of the ELISA plate (Maxisorb, Nunc, Thermo Fisher Scientific, USA) in coating buffer (PBS-7.2 pH and kept overnight at 4°C. The next day, the ELISA plate was incubated at 37°C, 150× *g* for 1 h. Further washing and blocking were done with wash buffer (Phosphate-Buffered saline-Tween 20) and blocking buffer (5% Skim Milk powder, 3% Lactalbumin hydrolysate, and 0.1% Tween-20), respectively. For each antigen concentration *Brucella* positive and negative samples were titred along the column of the plate (1 in 100; 1 in 50; 1 in 25; and 1 in 12.5 in vertical directions). Further, blocked with blocking buffer for 1 h at 37°C. After washing, conjugate (50 uL of 1:6000 dilution of horseradish peroxidase-conjugated anti-bovine IgG) was added and incubated at 37°C for 1 h. The reaction was visualized by adding substrate (o-phenylenediamine dihydrochloride + 30% H_2_O_2_). Finally, the reaction was arrested using a stopping solution (1M H_2_SO_4_). The OD was read at 492 nm by ELISA reader (Teccan, Switzerland).

### Evaluation of the suitability of recombinant protein I-ELISA

After optimizing the antigen concentration and serum dilution, ELISA was carried out on 113 each *Brucella* antibodies positive and negative cattle sera.

### Statistical analysis

Following standard definitions, a 2×2 contingency table and receiver operating characteristic (ROC) curve analysis were used to calculate all the diagnostic accuracy measures and confidence intervals based on OD values of the ELISA. The Youden’s index was employed for the analysis. The accuracy measures were expressed as DSe and DSp. Fleiss kappa (k) statistics was used to assess the agreement between two tests, where a value of k>0.75 is considered excellent, k=0.40-0.75 is considered fair to good, and k<0.40 is considered marginal to poor. p<0.05 was considered statistically significant for proportional analyses.

For the ELISA which has shown higher DSe, DSp, and area under curve (AUC) with a particular antigen, percent positivity (PP) {(Sample OD/Positive OD)×100} values were calculated. Further for the ELISA with the same antigen, other measures such as positive predictive value (PPV), negative predictive value (NPV), positive likelihood ratio, and negative likelihood ratio were calculated at different assumed prevalence levels (5%, 10%, and 20%).

All the statistical analyses were carried out using MedCalc, version 17.7.2 (MedCalc Software Ltd, Belgium).

## Results

### Selection of immunodominant proteins for expression

Out of 15 proteins identified, ten proteins were selected further for expression. On BLASTP analysis, five proteins were eliminated due to their marked amino acid sequence identity (>60%) with other pathogens (Table-3).

**Table-3:** Immunodominant proteins of *Brucella* *abortus* analyzed for identity by BLASTP.

S. No.	Protein	*Yersinia enterocolitica* (%)	*Salmonella enterica* (%)	*Vibrio cholerae* (%)	*Francisella tularensis* (%)	*Escherichia coli* (%)	*Stenotrophomonas maltophilia* (%)	Any other identity with other pathogens (%)
1.	SodC	60	60	53	53	52	34	-
2.	Serine protease	39	39	41	-	37	39	-
3.	BAB1-1885	-	-	-	-	-	60	-
4.	Twin-arginine		26			27	26	-
5.	BP26	29	28	-	-	-	41	-
6.	*Solute-binding family 5 protein	37	39	39	32	36	-	*Haematobacter* sp. (67%)
7.	*Leu/Ile/Val-binding family protein	23	23	-	-	23	-	*Bordetella bronchiseptica* (76%)
8.	*Branched chain amino-acid ABC transporter substrate-binding protein	-	27	-	-	-	-	*Bordetella* sp. (62%)
9.	Thiamine transporter	52	58	51	-	58	-	-
10.	Invasion protein B	-	-	-	-	-	-	-
11.	Brucella Lumazine synthase	38	56	32	-	51	34	-
12.	Bacterioferritin	50	52	52	-	52	54	-
13.	*Malate dehydrogense	33	32	30	64	39	-	*Haematobacter* sp. (77%)
14.	VirB12	-	34	-	-	32	42	-
15.	*Aldehyde dehydrogenase	30	31	31	54	31	54	*Bordetella* sp. (69%)

* Not selected for further expression. E values are less than 0; Sequence identity with *Ochrobactrum* sp. and Rhizobiales were not considered as they are generally soil organisms

### Expression and characterization of immunodominant proteins

**Figure-1:**
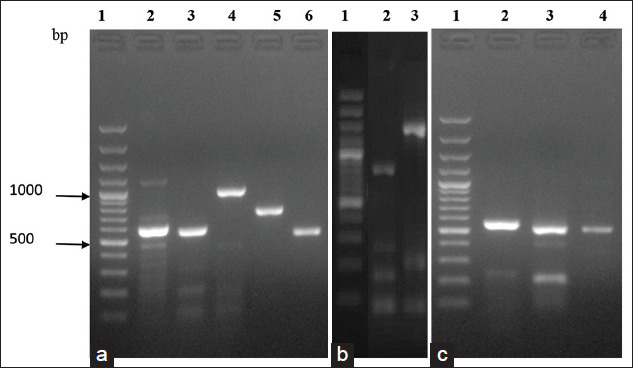
Agarose gel electrophoresis showing PCR amplicons of selected genes from *Brucella abortus* S99 DNA. (a) Lane 1: 100bp plus DNA ladder (Thermo Scientific); Lane 2: *twin arginine* gene (564 bp); Lane 3: *invB* gene (528 bp); Lane 4**:** t*hiB* gene (933 bp); Lane 5**:** *bp26* gene (666 bp); Lane 6: *sodC* gene (462 bp). (b) Lane 1: 100bp plus DNA ladder (Thermo Scientific);; Lane 2: *bab-1885* gene (1371 bp); Lane 3: *serine protease* gene (1464 bp). (c) Lane 1: 100bp plus DNA ladder (Thermo Scientific); Lane 2: *virB12* gene (513 bp); Lane 3: *bfr* gene (480 bp); Lane 4: *bls* gene (471 bp).

DNA extracted from *B. abortus* S99 was used to successfully amplify the target genes (Figure-1), cloned into pETite N-His Kan vector, and transformed into competent HI-Control 10G cells. Plasmid extracted from overnight grown colonies of HI-Control 10G clones containing the respective gene inserts ranged from 102.7 to 191.1 ng/μL which were optimal in their concentration for further subjecting to sequencing. Sequencing by vector-specific primers revealed the nucleotide sequences of the respective insert gene. All the sequences have shown 100% identity with *B. abortus* bv. 1 str. 9-941 DNA sequence. After confirmation of sequence identity, the plasmids of pETite N-His Kan vector containing individual inserts of *twin-arginine, invB*, *thiB*, *bp26*, *sodC*, *bab-1885*, *serine protease*, *virB12*, *bfr*, and *bls*, of the *B. abortus* S99 strain were successfully transformed into HI-Control BL21 (DE3) chemically competent cells, evident from the colony PCR with respective band size amplification for screened clones. The yield of the recombinant proteins ranged between 0.029 and 0.260 mg/mL.

**Figure-2:**
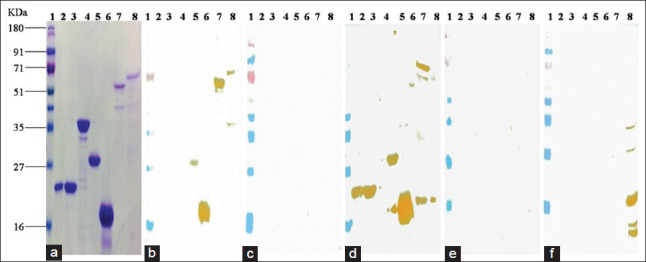
(a) SDS PAGE analysis of purified recombinant proteins. (b) Western blot analysis of purified recombinant proteins with *Brucella* antibodies positive cattle serum. (c) Western blot analysis of purified recombinant proteins with *Brucella* antibodies negative cattle serum. (d) Western blot analysis of purified recombinant proteins with *Brucella* S99 antibodies positive rabbit serum. (e) Western blot analysis of purified recombinant proteins with *Brucella* antibodies negative rabbit serum. (f) Western blot analysis of purified recombinant proteins with *Yersinia enterocolitica* O:9 positive rabbit serum. Lane 1: Prestained marker; Lane 2: Twin arginine; Lane 3: Invasion B; Lane 4: Thiamine transporter; Lane 5: BP26; Lane 6: SodC; Lane 7: BAB1-1885; Lane 8: Serine protease.

**Figure-3:**
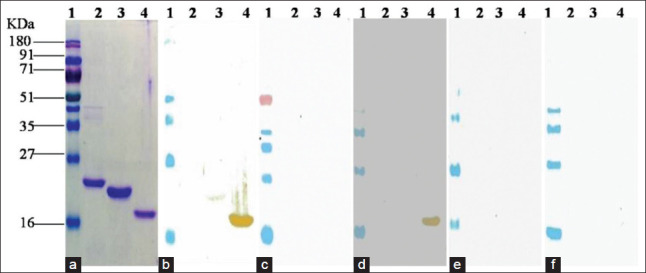
(a) SDS PAGE analysis of purified recombinant proteins. (b) Western blot analysis of purified recombinant proteins with *Brucella* antibodies positive cattle serum. (c) Western blot analysis of purified recombinant proteins with *Brucella* antibodies negative cattle serum. (d) Western blot analysis of purified recombinant proteins with *Brucella* S99 antibodies positive rabbit serum. (e) Western blot analysis of purified recombinant proteins with *Brucella* antibodies negative rabbit serum. (f) Western blot analysis of purified recombinant proteins with *Yersinia enterocolitica* O:9 positive rabbit serum. Lane 1: Prestained marker; Lane 2:VirB12; Lane 3: Bfr; Lane 4: BLS.

Western blot analysis of purified recombinant proteins with *Brucella* antibodies positive cattle serum has shown reactivity against BP26, SodC, BAB1-1885, Serine protease, Bfr, and BLS proteins. Twin arginine, InvB, BP26, SodC, BAB1-1885, Serine protease, and BLS proteins reacted with B*. abortus* S99 antibodies positive rabbit serum. None of the proteins reacted with *Y. enterocolitica* O:9 antibodies positive rabbit serum, *Brucella* antibodies negative cattle and rabbit serum (Figures-2 and 3).

### Evaluation of the suitability of expressed proteins for the screening of cattle brucellosis

**Figure-4:**
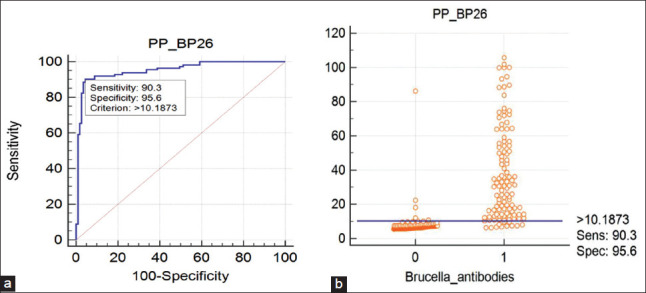
(a) ROC graph analysis of PP values of BP26 in ELISA with *Brucella* antibodies positive and negative cattle serum to optimize sensitivity and specificity. (b) Dot plot graph showing PP values of BP26 in ELISA with *Brucella* antibodies positive and negative cattle serum with cut off obtained from ROC curve analysis.

The proteins which were reactive with *Brucella* antibodies positive cattle serum in Western blot were subjected to I-ELISA. The checkerboard titration revealed an optimum antigen concentration of 0.5 mg/well for BP26, SodC, Bfr, Serine protease, 4 mg for BLS, and 8 mg for BAB1-1885 and serum dilution of 1:50 irrespective of the antigens. ELISA with recombinant proteins BP26, SodC, BAB1-1885, Serine protease, Bfr, and BLS in their optimized concentration with 113 *Brucella* antibodies positive and 113 negative field cattle serum samples and the ROC curve analysis revealed the cutoff along with DSe and DSp (Table-4). Considering all the proteins, BP26 based ELISA (Figure-4) has given a better AUC, DSe, and DSp, and hence PP values were calculated for ELISA with BP26 antigen which resulted in AUC of 0.953 (0.917-0.977), Youden index J of 0.8584, DSe of 90.27% and DSp of 95.58%. The cutoff based on PP value is 10.187 and the indicators of validity and utility were presented in Table-5. The Kappa statistic revealed an excellent agreement of 0.850 (0.781-0.918 at 95% CI) with the standard error of 0.0351.

**Table-4:** DSe and DSp of various recombinant proteins based enzyme-linked immune sorbent assay for serodiagnosis of cattle brucellosis.

Antigen	DSe%	DSp%	Area under curve	Standard Error	95% Confidence interval	z statistic	Significance level P	Youden index J
rBP26	92.04	96.46	0.968	0.0111	0.936-0.987	42.319	<0.0001	0.8850
rSodC	76.11	57.52	0.676	0.0360	0.610-0.736	4.869	<0.0001	0.3363
rBAB1-1885	54.87	69.91	0.603	0.0380	0.536-0.667	2.706	0.0068	0.2478
rHtrA	54.87	62.83	0.599	0.0375	0.532-0.664	2.641	0.0083	0.1770
rBfr	37.17	72.57	0.528	0.0385	0.461-0.595	0.737	0.4608	0.09735
rBLS	82.30	65.49	0.750	0.0331	0.688-0.805	7.564	<0.0001	0.4779

DSe: Diagnostic sensitivity, DSp: Diagnostic specificity

**Table-5:** Parameters of BP26 based ELISA for diagnosis of bovine brucellosis under various assumed prevalence level.

Assumed prevalence (%)	DSe%	CI%	DSp%	CI%	AUC	CI	LR+	CI	LR-	CI	PPV%	CI%	NPV%	CI%
5	90.27	83.25 to 95.04	94.69	88.80 to 98.03	0.92	0.88 to 0.96	17.00	7.79 to 37.12	0.10	0.06 to 0.18	47.22	29.07 to 66.14	99.46	99.06 to 99.69
10	90.27	83.25 to 95.04	94.69	88.80 to 98.03	0.92	0.88 to 0.96	17.00	7.79 to 37.12	0.10	0.06 to 0.18	65.38	46.38 to 80.49	98.87	98.03 to 99.35
20	90.27	83.25 to 95.04	94.69	88.80 to 98.03	0.92	0.88 to 0.96	17.00	7.79 to 37.12	0.10	0.06 to 0.18	80.95	66.06 to 90.27	97.49	95.68 to 98.56

DSe=Diagnostic Sensitivity, DSp=Diagnostic Specificity, CI=Confidence interval, AUC=Area under curve, LR+=Positive Likelihood ratio, LR- =Negative Likelihood ratio, PPV=Positive predictive value, NPV=Negative predictive value

## Discussion

In this study, an attempt was made to evaluate the suitability of immunodominant recombinant proteins that were not reactive to *Y. enterocolitica* O:9 antibodies for the diagnosis of cattle brucellosis. The immunoproteomics approach could be one of the efficient methods to identify specific immune-proteins among the greater range of proteins expressed by *Brucella* spp. [12]. Understanding the humoral immune response during *Brucella* infections will aid in the identification of immunogenic proteins. Appropriate identification and characterization of several immunoreactive proteins concerning seroreactivity can result in a satisfactory diagnostic test [13]. Although individual recombinant proteins have been described by many researchers for the diagnosis of brucellosis, a methodical way of comparing various immunodominant proteins and identification of best among them was undertaken in this study.

Using bovine sera, Ko *et al*. [14] identified immunodominant proteins of *B. abortus* 1119-3 strain, Kim *et al*. [15] identified that of *B. abortus* RB51 and Lee *et al*. [16] identified that of *B. abortus* 544. Lee *et al*. [17] used mice sera for *B. abortus* 544. From these studies, 61 *Brucella* proteins which are reactive only with *Brucella* antibodies and not with any other cross-reacting antibodies, in particular antibodies of *Y. enterocolitica* O:9 were identified. Further with the criteria of the outer membrane proteins (OMPs) or periplasmic proteins as more quantity of antibodies is expected against OMPs, the list was narrowed down to 11 proteins. However, since *Brucella* is an intracellular organism, the host immune response is not restricted to surface proteins and cytoplasmic proteins can also induce a higher antibody response [18], other proteins that were documented earlier were also considered with the idea of not missing out the immunodominant proteins irrespective of OMP or cytosolic. Besides, proteins such as chaperonin GroEL, Ribosomal protein L7/L12, chaperonin GroES [12], and OMP25 [14] were not considered due to their reactivity with cross-reacting antibodies. Letesson *et al*. [18] reported that OMP10, OMP16, OMP19, OMP25, OMP36, p15, and p39 were not useful for detecting naturally infected cattle. Thus, a meticulous selection of immunodominant proteins of *B. abortus* was made and further narrowed down based on the sequence identity with other pathogens.

In the case of allergic proteins, a minimum 35% shared identity, plus a minimum of 80 amino acid overlap length, are the criteria to identify sequences that have potential cross-reactivity between an unknown and a known allergen [19]. SodC, an immunodominant *Brucella* protein identified by researchers [14-16] has shown non-reactivity with *Y. enterocolitica* O:9 antibodies even though the sequence identity with the organism is 60%. Hence, in the case of proteins identified in this study, the identity threshold was fixed as 60% and above which if there is any sequence identity, those proteins were not considered further.

*B. abortus* strain 99 was used in this study as OIE recommends this strain [20] for the production of antigens for the diagnostic tests. Moreover, the amino acid sequences of proteins selected in this study are identical among various strains of *B. abortus*.

The selected proteins (n=10) were all expressed using pETite N-His Kan vector and as much of the earlier literature suggested that expression of BP26 and few other proteins leads to inclusion body formation, in this study directly after expression denaturing method of purification was carried out irrespective of whether the expressed proteins form inclusion bodies or not. There is a possibility for binding background contaminants under native conditions than under denaturing conditions and thus denaturing method of purification helped in yielding pure proteins. Cloeckaert *et al*. [21] observed a double band for rBP26 against infected sheep sera which could be due to the presence of both preprotein with its signal peptide and cleaved protein without signal peptide. The proteins in this study were expressed without signal sequences.

In Western blot, the additional reactivity of Twin arginine, InvB in rabbit hyperimmune serum compared to the cattle serum in this study might be due to the strong immune response in rabbits by use of adjuvants and frequent administration of killed antigen. The variations in the reactivity can be attributed to the differences like the serum used in the studies such as quantity and duration of exposure to the *Brucella* organism and stage of the infection. The observation of reactivity of Bfr only in cattle sera and not in rabbit hyperimmune serum needs to be explored further whether this reactivity occurs only in case of sera from infected and not from sera raised against the killed antigen. In addition, non-reactivity of these expressed antigens against *Y. enterocolitica* O:9 serum of rabbit confirms the right selection of candidate proteins for increasing the specificity of brucellosis diagnosis.

In absence of a gold standard, for the right classification of sera into *Brucella* antibodies positive and negative, a battery of tests/assays is required. Hence, in this study, RBPT, I-ELISA, and C-ELISA have been employed to select a panel of *Brucella* antibodies positive and negative sera for evaluating recombinant proteins. A test/assay with a high DSe is useful for “ruling out” a disease if an animal tests negative and a test with a high DSp is useful for “ruling in” a disease if an animal, tests positive. As LPS/whole antigen-based tests were more sensitive and less specific, the test which can contribute to more specificity and nominal sensitivity can provide additional information or can complement the existing test. In this study, the decreasing order of DSe was observed with BP26, BLS, SodC, BAB-1885, Serine protease, and Bfr based ELISA whereas the decreasing order of DSp was observed with BP26, Bfr, BAB-1885, BLS, and SodC based ELISA. DSe of 70.7%-80.5% and DSp of 84.6%-100% [7] DSe of 88.7%, DSp of 93.8% [22], DSe of 96.7%, and DSp of 95.4% [23] in bovines were observed in ELISA based on BP26 antigen; whereas, in this study, DSe of 92.04% and DSp of 96.46% were observed. The differences in DSe and DSp from different studies may be attributed to differences in samples under study, the gold standard against which the test was compared apart from inherent changes in the test procedure adopted. Youden’s index combines DSe and DSp and provides a single value for the comparison. Based on Youden’s index the performance of the BP26 is superior which is followed by BLS, SodC, and the rest of the proteins.

The AUC, DSe, DSp, and Youden’s index based on PP values of BP26 based ELISA were 0.953, 90.27%, 95.58%, and 0.8584 as against based on OD values 0.968, 92.04%, 96.46%, and 0.8850 (Table-4), respectively. Although there is a marginal reduction in all the values based on PP values in comparison to OD values, the calculations based on PP values are more stable. As the DSe is 90.27%, ruling in the individual animal may not be appropriate whereas it can be used as a test for detecting infected herds. PPV and NPV are both dependent on the prevalence of the disease and hence they were not calculated at the preliminary level of selection of the antigen because the number of positive and negative samples was pre-decided in the experiment and not indicative of prevalence level. Moreover, as PPV and NPV are dependent on prevalence, the values change concerning prevalence of the study area and not constant. Hence, AUC, DSe, DSp were considered in identifying the best antigen for ELISA as they do not vary with the prevalence level. However, PPV and NPV at assumed prevalence levels were calculated to assess the utility of the test. The NPV remains high and constant across various prevalence levels of 20%, 10%, and 5% from 97.49% to 99.46% whereas PPV increases from 47.22% to 80.95% from low prevalence (5%) to high prevalence (20%). Furthermore, the LR provides a suitable summary measure, which is independent of prevalence.

BP26 is highly conserved in the genus *Brucella*, and the recombinant protein from one *Brucella* species might be used for the serological diagnosis of infections caused by all the *Brucella* species, provided that the animal host can induce an immune response against this antigen [24]. Letesson *et al*. [18] documented that antibody responses to proteins were found to be limited to animals that developed an active brucellosis infection and this can be validated further with BP26 ELISA to identify active shedders of *Brucella* organisms in a herd. In this study, one of the important cross-reacting organism which has created trouble in few of the countries of the world, namely, *Y. enterocolitica* O:9 was addressed through the selection of *Brucella* antibodies reacting recombinant proteins but not reacted to experimentally raised hyperimmune rabbit serum to *Y. enterocolitica* O:9. Further, the identified recombinant protein should also be checked against other cross-reacting antibodies to ensure maximum specificity.

## Conclusion

As there are many claims for various recombinant proteins as the best recombinant proteins for diagnosing cattle brucellosis, this study has brought out the ranking and identified BP26 as the best suitable recombinant protein among them. BP26 based ELISA can be used as a screening test for detecting infected herds, to rule out cross-reactivity due to *Y. enterocolitica* O:9 in infected herds. It can also complement the existing diagnostic tests such as RBPT and sLPS based ELISA.

## Authors’ Contributions

MN conceived, designed, carried out the experiment, and wrote the draft of the manuscript. TJB and SSK assisted in carrying out the experiment. RS, GBMR, and JJK assisted in sample collection and other biological collection. MN and VB analyzed data. RKG, BRS, HR, and PR provided guidance and support to carry out the experiments. All authors read and approved the final manuscript.

## References

[1] Whatmore A.M, Koylass M.S, Muchowski J, Edwards-Smallbone J, Gopaul K.K, Perrett L.L (2016). Extended multilocus sequence analysis to describe the global population structure of the genus *Brucella*:Phylogeography and relationship to biovars. Front. Microbiol.

[2] Ducrotoy M.J, Conde-Álvarez R, Blasco J.M, Moriyón I (2016). A review of the basis of the immunological diagnosis of ruminant brucellosis. Vet. Immunol. Immunopathol.

[3] Al Dahouk S, Sprague L.D, Neubauer H (2013). New developments in the diagnostic procedures for zoonotic brucellosis in humans. Review. Rev. Sci. Tech.

[4] Adone R, Pasquali P (2013). Epidemiosurveillance of brucellosis. Rev. Sci. Tech.

[5] Hilbink F, Fenwick S.G, Thompson E.J, Kittelberger R, Penrose M, Ross G.P (1995). Non-specific seroreactions against *Brucella abortus* in ruminants in New Zealand and the presence of *Yersinia enterocolitica* 0:9. N. Z. Vet. J.

[6] Nagaraju N.R, Isloor S, Rao S.M, Rajasekhar M (2003). Seroprevalence of bovine yersiniosis in India. Indian J. Anim. Sci.

[7] Munoz P.M, Marin C.M, Monreal D, Gonzalez D, Garin-Bastuji B, Diaz R, Mainar-Jaime R.C, Moriyon I, Blasco J.M (2005). Efficacy of several serological tests and antigens for diagnosis of bovine brucellosis in the presence of false-positive serological results due to *Yersinia enterocolitica* O:9. Clin. Diagn. Lab. Immunol.

[8] Godfroid J, Kasbohrer A (2002). Brucellosis in the European Union and Norway at the turn of the twenty-first century. Vet. Microbiol.

[9] Alton G.G, Jones L.M, Angus R.D, Verger J.M (1988). Techniques for the Brucellosis Laboratory. Institute National De La Recherche Agronomique, Paris, France.

[10] World Organization for Animal Health (OIE). Principles and methods of validation of diagnostic assays for infectious diseases (2016). In:Manual of Diagnostic Tests and Vaccines for Terrestrial Animals. Ch. 1.1.2. OIE, Paris.

[11] Crowther J.R (2009). The ELISA Guidebook. Methods in Molecular Biology.

[12] Al Dahouk S, Nockler K, Scholz H.C, Tomaso H, Bogumil R, Neubauer H (2006). Immunoproteomic characterization of *Brucella abortus*1119-3 preparations used for the serodiagnosis of *Brucella* infections. J. Immunol. Methods.

[13] Pajuaba A.C, Silva D.A, Almeida K.C, Cunha-Junior J.P, Pirovani C.P, Camillo L.R, Mineo J.R (2012). Immunoproteomics of *Brucella abortus* reveals differential antibody profiles between S19-vaccinated and naturally infected cattle. Proteomics.

[14] Ko K.Y, Kim J.W, Her M, Kang S.I, Jung S.C, Cho D.H, Kim J.Y (2012). Immunogenic proteins of *Brucella abortus* to minimize cross-reactions in brucellosis diagnosis. Vet. Microbiol.

[15] Kim J.Y, Sung S.R, Lee K, Lee H.K, Kang S.I, Lee J.J, Jung S.C, Park Y.H, Her M (2014). Immunoproteomics of *Brucella abortus* RB51 as candidate antigens in serological diagnosis of brucellosis. Vet. Immunol. Immunopathol.

[16] Lee J.J, Simborio H.L, Reyes A.W, Kim D.G, Hop H.T, Min W, Her M, Jung S.C, Yoo H.S, Kim S Proteomic analyses of the time course responses of mice infected with *Brucella abortus* 544 reveal immunogenic antigens FEMS Microbiol. Lett.

[17] Lee J.J, Simborio H.L, Reyes A.W, Kim D.G, Hop H.T, Min W, Her M, Jung S.C, Yoo H.S, Kim S (2015). Immunoproteomic identification of immunodominant antigens independent of the time of infection in *Brucella abortus* 2308-challenged cattle. Vet. Res.

[18] Letesson J.J, Tiber A, Van-Eynde G, Wansard V, Weynants V, Denoel P, Saman E (1997). Humoral immune responses of *Brucella*-infected cattle, sheep and goats to eight purified recombinant *Brucella* proteins in an indirect enzyme-linked immunosorbent assay. Clin. Diagn. Lab. Immunol.

[19] McClain S (2017). Bioinformatic screening and detection of allergen cross-reactive IgE-binding epitopes. Mol. Nutr. Food Res..

[20] World Organization for Animal Health (OIE). (2016a) Brucellosis (*Brucella abortus B. melitensis*and *B. suis*) (infection with *B. abortus, B. melitensis* and *B. suis*) In:Manual of Diagnostic Tests and Vaccines for Terrestrial Animals. Ch. 2.1.4. OIE, Paris.

[21] Cloeckaert A, Debbarh H.S, Vizcaíno N, Saman E, Dubray G, Zygmunt M.S (1996). Cloning, nucleotide sequence, and expression of the *Brucella melitensis* bp26 gene coding for a protein immunogenic in infected sheep. FEMS Microbiol. Lett.

[22] Chaudhuri P, Prasad R, Kumar V, Gangaplara A (2010). Recombinant OMP 28 antigen-based indirect ELISA for serodiagnosis of bovine brucellosis. Mol. Cell. Probes.

[23] Lim J.J, Kim D.H, Lee J.J, Kim D.G, Min W, Lee H.J, Rhee M.H, Chang H.H, Kim S (2012). Evaluation of recombinant 28 kDa outer membrane protein of *Brucella abortus* for the clinical diagnosis of bovine brucellosis in Korea. J. Vet. Med. Sci.

[24] Seco-Mediavilla P, Verger J.M, Grayon M, Cloeckaert G.A, Marín C.M, Zygmunt M.S, Fernández-Lago L, Vizcaíno N (2003). Epitope mapping of the *Brucella melitensis* BP26 immunogenic protein:Usefulness for diagnosis of sheep brucellosis. Clin. Diagn. Lab. Immunol.

